# Leprosy

**Published:** 1898-05

**Authors:** 


### EDITORIAL DEPARTMENT.

F. E. DANIEL, M. D., Editor.
S. E. HUDSON, M. D., Managing Editor.
A. J. SMITH. M. D., Galveston, Associate Editor.

Published Monthly at Austin, Texas, by Drs. Daniel and Hudson. Subscription price $2.00 a year in advance.

Eastern Representatives: Messrs. Monihan & Fairchild, St. Paul Building, 220 Broadway, New York City.

Official organ of the West Texas Medical Association, the Houston District Medical Association, the Austin District Medical Society, the Brazos Valley Medical Association, the Galveston County Medical Society, and several others.

### LEPROSY.

This ancient and historical disease is just now exciting unusual interest, both in the medical press and in the newspapers throughout the country. This increased interest is due to the prospective annexation by the United States of the Hawaiian Islands, where the disease prevails to a considerable extent, there being at this time about one thousand lepers on these islands.

For this reason the annexation of the islands has been strongly opposed by some of our statesmen, who fear the further extension of the disease. We think, however, that the apprehension is without foundation, as the government of the islands (which is now a republic) has already taken steps to "stamp out" the disease by complete separation of the sick from the well, and segregating or colonizing the former, and thus forcing the lepers to intermarry with each other, if at all. By this means it is hoped that by a process of rapid degeneration the race of lepers may soon become extinct. Some difficulty, however, has been experienced in carrying out this plan, on account of family ties, and the strong attachments of friendship.

The origin of the disease is involved in doubt and mystery. We believe, however, that there is a general census of opinion amongst medical writers that leprosy and syphilis are near of kin, and that the former is in fact a modification of the latter.

The history of leprosy may be traced far back into antiquity. It has been well described by the earlier medical authors, including Hippocrates, who lived in the fifth century before

Christ. We have no doubt but the Israelitish king, David, from his symptoms, as detailed by himself, suffered either from leprosy or tertiary syphilis. The writer of the book of Job also describes the disease with admirable fidelity, and we strongly suspect that the malady which Satan was permitted to lay upon Job was only syphilis, which as before stated, is probably as near of kin as first cousin to leprosy.

The disease is not, as has been supposed, indigenious to or confined to any particular locality, or part of the earth's surface; neither is any race of people more liable to it than any other race, except on account of environmental circumstances. It may prevail in any country or amongst any people, if sanitary and hygienic precautions are neglected. The fact is well established that insanitary conditions, both public and private, favor the development and spread of the disease. It is universally admitted to be infectious and also hereditary. We are of opinion, however, that it is not intensely contagious, as it is a well known fact that well persons may live in the same house for years with the leprous without contracting the disease.

Its greater prevalence amongst savage and half civilized peoples is accounted for by neglect of sanitary conditions. The disease is gradually disappearing from European countries. In Great Britain, where it prevailed quite extensively a century or more ago, it has been completely extinguished. It still prevails, however, to a limited extent in Norway, Sweden and Turkey. In the United States, it is found only in Louisiana and Florida.

It is still largely prevalent in most Eastern countries, on the Asiatic continent, as well as on some of the islands of the far East. In fact, the original habitat of the disease was and is amongst the nations of the East.

Leprosy has often been confounded, and we think improperly, with psoriasis, but it is undoubtedly identical with some forms of elephantiasis.

Certain tissues of the body are more liable to attack by the disease, and of these the skin is first in order, especially the face, knees, elbows and backs of the hands. Second, the nerves which become thickened, irritated, and finally destroyed. On the skin hypertrophies, tuberosities and nodules are formed, which eventually become bleached, or whitened, in patches, and finally break down from a process of destructive ulceration.

In cases where the nerves are primarily affected, in course of time the morbid process extends to the cellular and muscular tissues, causing in some cases enormous hypertrophies. This class is known and designated as elephantiasis graecorum, and is not unknown in this State, at least two cases having been observed by the writer. If leprosy is indeed only one of the protean forms of syphilis, it is in some respects a departure from the ordinary forms of that disease. Syphilis is generally regarded as curable, while leprosy is universally admitted by medical men to be incurable. It is true that apparent cures occur, but it is claimed by those of greatest experience, that such are only periods of latency, or quiescence, which may last for years, but the disease always manifests its presence later in life. Therefore the treatment in the present state of our knowledge must be symtomatic or empirical.

